# Estimating Arctic Sea Ice Thickness with CryoSat-2 Altimetry Data Using the Least Squares Adjustment Method

**DOI:** 10.3390/s20247011

**Published:** 2020-12-08

**Authors:** Feng Xiao, Fei Li, Shengkai Zhang, Jiaxing Li, Tong Geng, Yue Xuan

**Affiliations:** Chinese Antarctic Center of Surveying and Mapping, Wuhan University, 129 Luoyu Road, Wuhan 430079, China; shaw89@whu.edu.cn (F.X.); zskai@whu.edu.cn (S.Z.); listening@whu.edu.cn (J.L.); t.geng@whu.edu.cn (T.G.); xuanyue@whu.edu.cn (Y.X.)

**Keywords:** Arctic, sea ice thickness, CryoSat-2, seasonal and annual variations, least squares adjustment

## Abstract

Satellite altimeters can be used to derive long-term and large-scale sea ice thickness changes. Sea ice thickness retrieval is based on measurements of freeboard, and the conversion of freeboard to thickness requires knowledge of the snow depth and snow, sea ice, and sea water densities. However, these parameters are difficult to be observed concurrently with altimeter measurements. The uncertainties in these parameters inevitably cause uncertainties in sea ice thickness estimations. This paper introduces a new method based on least squares adjustment (LSA) to estimate Arctic sea ice thickness with CryoSat-2 measurements. A model between the sea ice freeboard and thickness is established within a 5 km × 5 km grid, and the model coefficients and sea ice thickness are calculated using the LSA method. Based on the newly developed method, we are able to derive estimates of the Arctic sea ice thickness for 2010 through 2019 using CryoSat-2 altimetry data. Spatial and temporal variations of the Arctic sea ice thickness are analyzed, and comparisons between sea ice thickness estimates using the LSA method and three CryoSat-2 sea ice thickness products (Alfred Wegener Institute (AWI), Centre for Polar Observation and Modelling (CPOM), and NASA Goddard Space Flight Centre (GSFC)) are performed for the 2018–2019 Arctic sea ice growth season. The overall differences of sea ice thickness estimated in this study between AWI, CPOM, and GSFC are 0.025 ± 0.640 m, 0.143 ± 0.640 m, and −0.274 ± 0.628 m, respectively. Large differences between the LSA and three products tend to appear in areas covered with thin ice due to the limited accuracy of CryoSat-2 over thin ice. Spatiotemporally coincident Operation IceBridge (OIB) thickness values are also used for validation. Good agreement with a difference of 0.065 ± 0.187 m is found between our estimates and the OIB results.

## 1. Introduction

Sea ice forms an interface between the upper ocean and the atmosphere and plays an important role in the Earth’s climate and environmental system through modifying the radiative and energy balance and modulating the exchange of heat and moisture between the ocean and atmosphere [1]. Screen and Simmonds [2] noted that the Arctic sea ice reduction has had a leading role in recent Arctic temperature amplifications. To better understand the polar and global climate system and processes, long-term and accurate observations of Arctic sea ice are required.

Thickness is one of the most important sea ice parameters because its changes during the seasonal cycle may cause significant impacts on global change [3,4,5]. Sea ice thickness is sensitive to the coupling between the atmosphere and the ocean, which directly determines the exchange rate of energy and matter between them. Accurately obtaining the sea ice thickness and its changes not only helps in understanding the climate and environmental changes but also has a significant impact on maritime shipping.

Satellite altimeters are widely used to retrieve hemispheric estimates of sea ice thickness [6,7,8,9,10,11,12]. The altimetric sea ice thickness retrieval method relies on measurements of freeboard. The sea ice freeboard is defined as the height of sea ice surface above local sea level, and it is determined from a comparison of elevation measurements over sea ice and leads. Then, the freeboard estimates are converted to sea ice thickness based on the hydrostatic equilibrium equation, as a function of snow thickness, snow density, sea ice density, and sea water density. Therefore, the uncertainties in sea ice freeboard to thickness conversion are related to the knowledge about snow depth and sea ice density [13,14]. Zygmuntowska et al. [15] found that the uncertainty in snow depth contributes up to 70% of the total uncertainty, and the ice density contributes 30–35%. Values based on field measurements or numerical simulations are used in previous studies. For example, Laxon et al. [7] used a monthly climatology of snow depth (hereafter named W99) [16] in Arctic sea ice thickness retrieval with radar measurements from ERS-1 and ERS-2. W99 is based on measurements of drifting stations during the second half of the 20th century and is still used as the main source of snow depth and density data. However, analyses of snow radar data show that there are known differences between W99 and the current snow depth on younger Arctic sea ice [17]. Therefore, half of the snow depth from W99 is used in first-year ice (FYI) thickness retrieval [11,13]. In the case of sea ice density, previous studies have used a wide range of values. For example, in the study by Kwok et al. [9], an ice density of 925 kg/m^3^ was used, Kern et al. [18] used a value of 900 kg/m^3^, while Laxon et al. [11] used an estimate of 917 kg/m^3^ for FYI and 882 kg/m^3^ for multiyear ice (MYI). The range of sea ice density values from MYI is particularly large at 43 kg/m^3^, which can cause a bias of 1.1 m in sea ice thickness estimation [19].

The selection of snow depth and sea ice density plays important roles in the retrieval of sea ice thickness, and it also has implications for the uncertainty in temporal trends in Arctic sea ice thickness estimates [15]. The focus of this study is to minimize the uncertainties in sea ice thickness estimation. We develop a new method to convert sea ice freeboard to thickness without prior knowledge of snow depth and densities of snow, sea ice, and sea water. We derive estimates of sea ice thickness as well as sea ice density for the Arctic Ocean from CryoSat-2 radar altimeter measurements with the newly developed method. The sea ice thickness results are compared with existing CryoSat-2-based sea ice thickness products and spatiotemporally coincident Operation IceBridge (OIB) thickness products.

## 2. Data

### 2.1. CryoSat-2 Data

CryoSat-2 was launched in 2010 with the purpose of observing Earth’s cryosphere. CryoSat-2 carries a newly developed radar altimeter named the synthetic aperture radar (SAR) interferometric radar altimeter (SIRAL) [20]. SIRAL features high accuracy over ice margins and sea ice regions due to the combination of SAR and interferometry techniques. CryoSat-2 provides altimetry data up to a latitude of 88° S/N. SIRAL samples the surface every 300 m along the track. SIRAL also features narrow across-track spacing of 2.5 km at 70° S/N. This is an improvement compared to the coarse across-track spacing of 25 km at 70° S/N of previous radar altimeters.

We use CryoSat-2 Baseline-C L1b and L2 data available from the European Space Agency (ESA) to estimate the Arctic sea ice thickness. The L1b data contain the multi-looked echo power, measurement time, geolocation, satellite altitude, surface type flag, and other instrumental measurements. The product includes additional information, such as geophysical and tidal corrections and quality flags. The L2 data include surface elevation estimates.

### 2.2. CryoSat-2 Sea Ice Thickness Products

Recently publicly available sea ice thickness products based on CryoSat-2 have been provided by institutions, including the Centre for Polar Observation and Modelling (CPOM) [10,21], the Alfred Wegener Institute (AWI) [5], the NASA Goddard Space Flight Centre (GSFC) [19], the NASA Jet Propulsion Laboratory (JPL) [11], the ESA Climate Change Initiative (CCI) [22], and the Laboratoire d’Études en Géophysique et Océanographie Spatiales (LEGOS) Center for Topographic studies of the Ocean and Hydrosphere (CTOH) [23]. JPL provides CryoSat-2 thickness data from January 2011 to December 2015, CCI and CTOH provide thickness data from November 2011 to April 2017, while this study estimates the Arctic sea ice thickness from November 2010 to April 2019. For comparison, we utilize thickness products from AWI, CPOM, and GSFC, which cover the period from November 2010 to present.

CPOM provides monthly Arctic sea ice thickness product on a 5 km grid with CryoSat-2 altimetry data. CPOM also provides near-real-time Arctic sea ice thickness estimates based on fast-delivery CryoSat-2 data [24]. AWI provides sea ice thickness product on a 25 km grid. The AWI ice thickness product also includes other parameters, such as sea ice type, snow depth, and radar freeboard. GSFC provides 30 days of ice thickness estimates as well as freeboard estimates on a 25 km grid. The three sea ice thickness products are available for the months of October through April.

### 2.3. OIB Sea Ice Thickness Product

Operation IceBridge (OIB) is a NASA program of airborne remote sensing measurements designed to fill the gap in measurements between ICESat and ICESat-2. OIB can obtain sea ice thickness estimates by onboard instruments including the airborne topographic mapper and snow radar. Here, we employ the OIB sea ice thickness datasets available at the National Snow and Ice Data Center (NSIDC) [25] to validate our sea ice thickness results. The datasets consist of measurements from the Airborne Topographic Mapper laser altimeter, Digital Mapping System camera, and the University of Kansas’ 2–8 GHz snow radar. Sea ice thickness is available at a 40 m spatial sampling resolution along the flight lines.

## 3. Methodology

### 3.1. Sea Ice Freeboard Retrieval

Before performing the sea ice freeboard calculation, measurements of the leads and ice should be discriminated. Leads are openings in the ice that appear even in regions covered by thick ice [26]. In published articles on sea ice thickness estimation with CryoSat-2, the pulse peakiness (PP) and stack standard deviation (SSD) are widely used for lead detection. For SAR mode, echoes with a PP > 18 and an SSD < 6.29 are identified as leads, while for SARIn mode, echoes with a PP > 18 and an SSD < 4.62 are identified as leads [21].

The radar measurements are processed for each CryoSat-2 orbit. First, geoid undulations and the mean sea surface height (MSS) are removed by subtracting the MSS height ($h_{mss}$):

(1) $$h_{r}=h-h_{mss}$$

where *h* is the surface elevation and *h* together with $h_{mss}$ can be obtained using the L2 product. Then, the freeboard ($h_{fb}$) is obtained as follows [5]:

(2) $$h_{fb}=h_{r}-h_{ssha}$$

The local instantaneous sea surface height anomaly ($h_{ssha}$) can be derived from the lead elevations.

### 3.2. Sea Ice Thickness Estimation

Estimation of the snow-covered sea ice thickness from altimeter measurements assumes hydrostatic equilibrium. Once the sea ice freeboard ($h_{fb}$) is determined from altimeter data, the sea ice thickness ($h_{si}$) can be calculated by Equation (3) [5]:

(3) $$h_{si}=\frac{\rho_{sw}}{\rho_{sw}-\rho_{si}}h_{fb}+\frac{\rho_{s}-\rho_{sw}}{\rho_{sw}-\rho_{si}}h_{s}+\frac{\rho_{sw}}{\rho_{sw}-\rho_{si}}\theta \cdot h_{s}$$

where $h_{s}$ is the snow depth, θ is the penetration factor of CryoSat-2 radar signals, and $\rho_{sw}$, $\rho_{si}$, and $\rho_{s}$ are the densities of sea water, sea ice, and snow, respectively. To accurately estimate the sea ice thickness, the snow and penetration depths as well as the density parameters need to be observed concurrently with the altimeter measurements. However, this task is challenging due to the extreme weather conditions of the Arctic Ocean. Thus, previous studies have used typical values based on field measurements or numerical simulations. Table 1 shows some typical values used in past studies. For example, the snow depth simulated by the W99 climatology model has been widely applied. However, the W99 model was derived from in situ data collected over multiyear ice (MYI) from 1954 to 1991. Due to the large observed loss of multiyear ice in recent years, climatology may no longer provide an accurate representation of current snow conditions. Thus, some researchers have suggested that the snow depth on first-year ice (FYI) is approximately 50% of that given by W99 [5,10,27,28]. The sea ice density values used in previous studies varied from 882 to 915 kg/m^3^ [5,9,10,18,27,28,29,30,31,32], and the sea water density varied from 1024 to 1030 kg/m^3^ [10,18,24,29,31,33,34]. Snow density of 300 kg/m^3^ [32,35] and 320 kg/m^3^ [29,31,33] are used in some studies, while other studies [7,36] use density values from W99, which vary from 233 to 363 kg/m^3^.

To minimize the uncertainty from the input parameters ($h_{s}$, θ, $\rho_{sw}$, $\rho_{si}$, and $\rho_{s}$) for the sea ice thickness estimation, we first separate the calculated freeboard values into regularly spaced 5 km × 5 km geographical regions. Then, in each grid cell, we model the freeboard as a quadratic function of the local ice surface terrain:

(4) $$h_{fb}(x,y)=\bar{h_{fb}}+a_{0}x+a_{1}y+a_{2}x^{2}+a_{3}y^{2}+a_{4}xy$$

where $\bar{h_{fb}}$ indicates the mean freeboard of the grid cell and x and y represent the longitudinal and latitudinal surface distances between the observation and the central point of the grid cell, respectively. According to Equation (3),

(5) $$\bar{h_{fb}}\;=\;(1-\frac{\rho_{si}}{\rho_{sw}})\bar{h_{si}}+(1-\frac{\rho_{s}}{\rho_{sw}}-\theta )h_{s}$$

Thus, Equation (4) can be rewritten as follows:

(6) $$\begin{array}{ccc} h_{fb}(x,y) & = & (1-\frac{\rho_{si}}{\rho_{sw}})\bar{h_{si}}+(1-\frac{\rho_{s}}{\rho_{sw}}-\theta )h_{s}+a_{0}x+a_{1}y+a_{2}x^{2}+a_{3}y^{2}+a_{4}xy\\ & = & a_{0}x+a_{1}y+a_{2}x^{2}+a_{3}y^{2}+a_{4}xy+a_{5}\bar{h_{si}}+a_{6} \end{array}$$

where $a_{5}=1-\frac{\rho_{si}}{\rho_{sw}}$, $a_{6}=(1-\frac{\rho_{s}}{\rho_{sw}}-\theta )h_{s}$, and $\bar{h_{si}}$ is the mean sea ice thickness of the grid cell.

We can retrieve the model coefficients ($a_{0}$~$a_{6}$) and $\bar{h_{si}}$ in each grid cell using the least squares adjustment (LSA) method [38,39], which acquires good prior knowledge of the unknown parameters. A proper weighting scheme for the observations is needed for a successful solution.

Prior values for $a_{0}$~$a_{4}$ are set to zero. For $a_{5}$ and $a_{6}$, the prior values are set by referencing empirical values of the densities in the literature: $a_{5}$ is set to 0.12, and $a_{6}$ is set to –0.05. The prior value of the mean sea ice thickness ($\bar{h_{si}}$) is set to 1 m. The weighting scheme is based on the distance between the observation and the central point of the grid cell. The weight of a certain observation ($P_{i}$) is inversely proportional to the distance to the central point of the grid cell:

(7) $$P_{i}=\frac{1}{\mathrm{distance}([{\mathrm{lon}}_{i},{\mathrm{lat}}_{i}],[{\mathrm{lon}}_{0},{\mathrm{lat}}_{0}])}$$

where ${\mathrm{lon}}_{i}$ and ${\mathrm{lat}}_{i}$ are the locations of a certain observation, and ${\mathrm{lon}}_{0}$ and ${\mathrm{lat}}_{0}$ are the locations of the central point of the grid.

**Figure 1:**
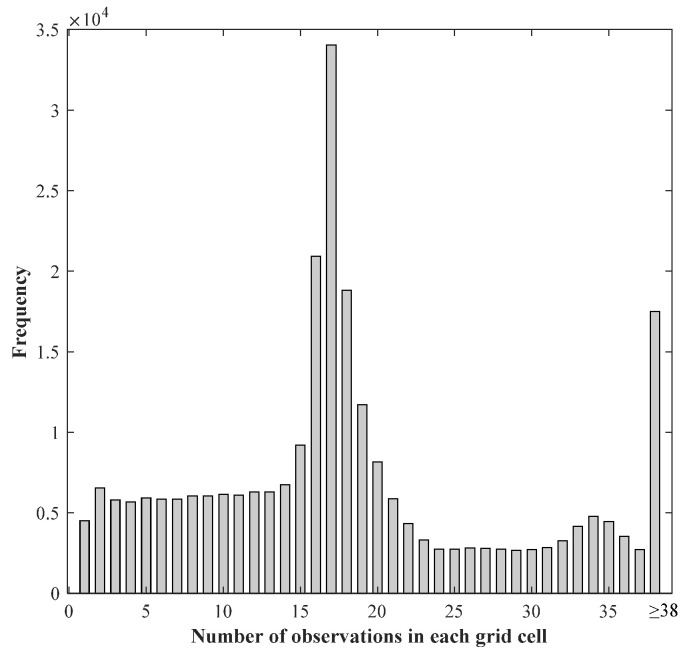
Frequencies of the numbers of observations in each grid cell.

To determine the eight unknown parameters, at least eight observations are needed in each grid cell. The numbers of observations that fall in each grid cell are calculated. Figure 1 shows the frequencies of the numbers of observations in each grid cell. Approximately 84.72% of grid cells have eight or more observations to constrain each solution. To fill data gaps in the 5 km grid, we duplicate the calculation with a 10 km resolution grid. Missing data in the 5 km grid are filled by the re-sampled 10 km grid. By using this model fit method, we are able to generate sea ice thickness estimates, which are not affected by the uncertainty in the snow depth and density parameters.

## 4. Results and Discussions

### 4.1. Arctic Sea Ice Thickness Results Derived from Least Square Adjustment Method

Based on the LSA method, we are able to derive estimates of the Arctic sea ice thickness for November 2010 through April 2019 using CryoSat-2 altimetry data. In this paper, the Arctic sea ice region is defined as latitudes above and including 65° N. We do not calculate the sea ice thickness in the months of May to September due to melt ponds. This is because during the melt season, it is difficult to distinguish between measurements from leads and ice floes due to melt ponds forming on the ice that cause specular return echoes [21,40]. Specular return echoes caused by melt ponds dominate the majority of return waveforms, and less than 1% of the waveforms are classified as ice floe returns.

**Figure 2:**
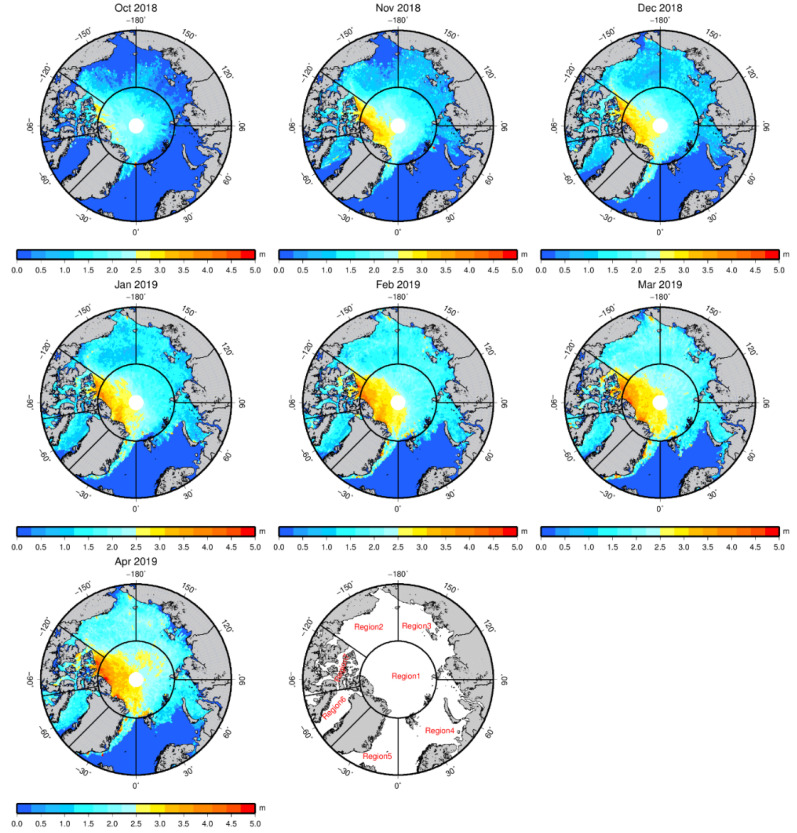
Arctic sea ice thickness distribution with the least squares adjustment (LSA) method for the 2018–2019 Arctic sea ice growth season from October to April. The thickness distribution is presented in seven regions, region 1: Arctic Ocean; region 2: Beaufort and Chukchi Seas; region 3: Laptev and East Siberian Seas; region 4: Kara and Barents Seas; region 5: Greenland Sea; region 6: Baffin Bay; and region 7: Canadian Arctic Archipelago.

**Figure 3:**
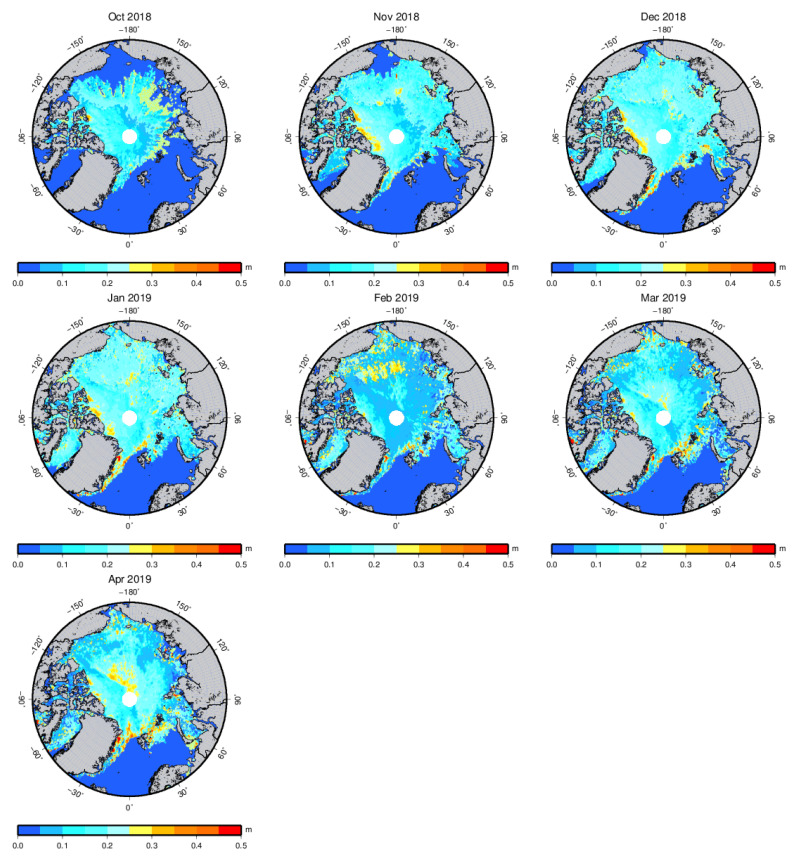
Arctic sea ice thickness estimation uncertainty with the LSA method for the 2018–2019 Arctic sea ice growth season from October to April.

Figure 2 shows an example of the spatial distribution of the sea ice thickness for the 2018–2019 Arctic sea ice growth season. Figure 3 shows the sea ice thickness uncertainty distribution with the LSA method for the 2018–2019 Arctic sea ice growth season from October to April. The thickness estimation uncertainty is calculated by the difference of $\bar{h_{si}}$ in the last two iterations. The estimation uncertainty is less than 0.25 m in most areas of the Arctic Ocean. Large uncertainty can be found near the coast and sea ice margins.

**Figure 4:**
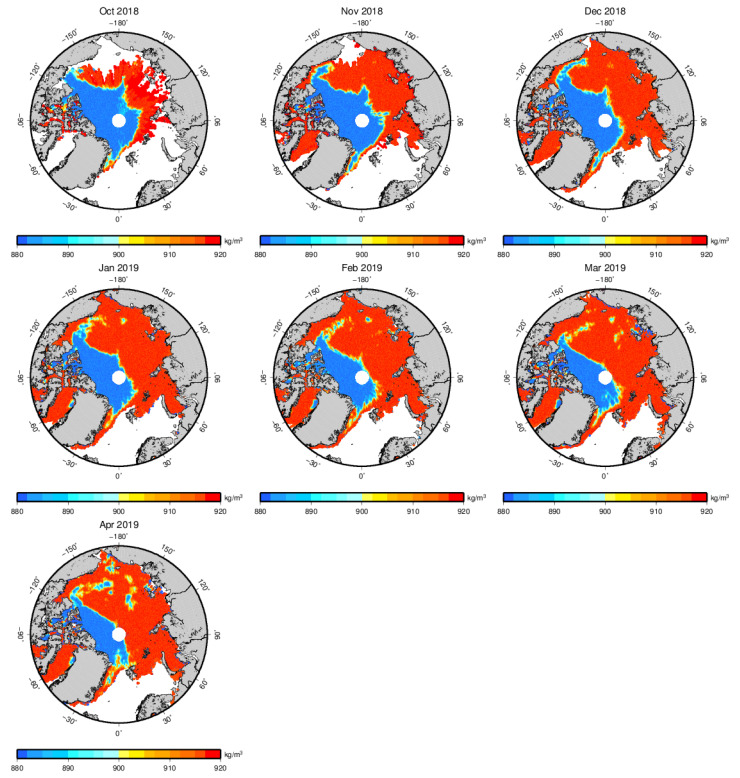
Arctic sea ice density with the LSA method for the 2018–2019 Arctic sea ice growth season from October to April.

We can also derive sea ice density from $a_{5}$ by setting sea water density as a fixed value (1024 kg/m^3^). Figure 4 shows the sea ice density distribution for the 2018–2019 Arctic sea ice growth season from October to April. It can be easily found that the thin ice density is larger than thick ice density. Thin ice density ranges from 915 to 920 kg/m^3^, while the thick ice density ranges from 880 to 885 kg/m^3^.

**Figure 5:**
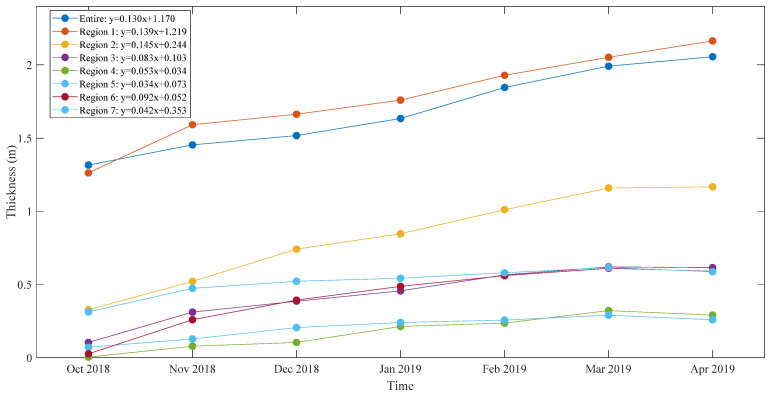
Mean sea ice thickness of the entire Arctic basin and seven regions for the 2018–2019 Arctic sea ice growth season.

The sea ice thickness results are presented in seven regions covering the Arctic Basin. The region-dividing criterion is based on Ye et al. [41]. Region 1 covers the Arctic Basin with latitudes over 80° N, region 2 includes the Beaufort and Chukchi Seas, region 3 includes the Laptev and East Siberian Seas, region 4 includes the Kara and Barents Seas, region 5 is the Greenland Sea, region 6 covers Baffin Bay, and region 7 covers the Canadian Arctic Archipelago. Table 2 and Figure 5 show the mean sea ice thicknesses of the entire Arctic Basin and seven regions during the 2018–2019 Arctic sea ice growth season.

During the 2018–2019 sea ice growth season, the thickness growth rate of the entire Arctic Basin is approximately 13 cm per month. In October, the sea ice thickness is the smallest, with an average value of 1.315 m, and a few thick ice packs can be seen off the northern coast of the Canadian Archipelago. Afterward, the sea ice thickness increases over the course of the sea ice growth season. In November, more thick ice appears in region 1 and north of region 7, and thin ice begins to appear in the south of regions 2, 3, 4, and 6. In December, the ice thickness growth rate is small (6.4 cm per month), and more thin ice appears in the southwest of region 2. In January, more thick ice occurs in region 1. In February, the ice thickness experiences the largest growth rate (21.2 cm per month). In March, the thick ice area continues to expand. In April, the ice growth rate decreases to 6.5 cm per month, but more thick ice appears in the eastern part of region 1.

Region 1 features the largest mean ice thickness (1.775 m) and the second highest growth rate (13.9 cm per month) during the 2018–2019 sea ice growth season. The largest ice growth rate in region 1 occurs in November (32.9 cm per month). Region 2 features the highest ice growth rate (14.5 cm per month) and the second largest mean ice thickness (0.825 m). In region 2, the ice growth rate from October to December reaches 20.7 cm per month, which is much larger than the rate during the following months (11.7 cm per month). The ice thickness growth rates in region 3 and region 6 are very close. Region 4 has the smallest mean ice thickness (0.178 m), and region 5 has the lowest ice growth rate (3.4 cm per month). The ice thickness in regions 3, 4, 5, 6, and 7 decreases in April, while it continues to grow in the other regions. Owing to the growth in region 1 and region 2, the mean ice thickness in the entire Arctic Basin shows a small increase in April.

### 4.2. Arctic Sea Ice Thickness Variations

**Figure 6:**
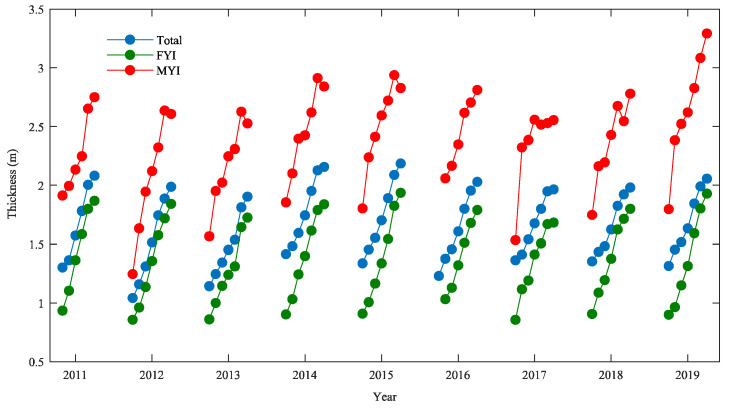
Monthly average thickness variations of the Arctic sea ice from November 2010 to April 2019 estimated using the LSA method. The blue dots indicate total ice thickness, the green dots indicate first-year ice thickness, and the red dots indicate multiyear ice thickness.

Monthly average thickness variations of the Arctic sea ice derived with the LSA method are shown in Figure 6. Seasonal variations can be clearly found in the Arctic sea ice thickness. The total ice thickness increases each month over a given sea ice growth season from October until April, which is also generally true for the thickness of FYI and MYI. In some years (e.g., 2012, 2013, 2014, 2015), the MYI thickness drops slightly from March to April due to the onset of summer melt. The variations of total ice thickness are close to the variations of FYI thickness with a correlation coefficient of 0.916 (0.817 for MYI thickness versus total ice thickness), which indicates that FYI is the dominant ice type, because the MYI extent is now less than one-third of the Arctic Ocean [42].

**Figure 7:**
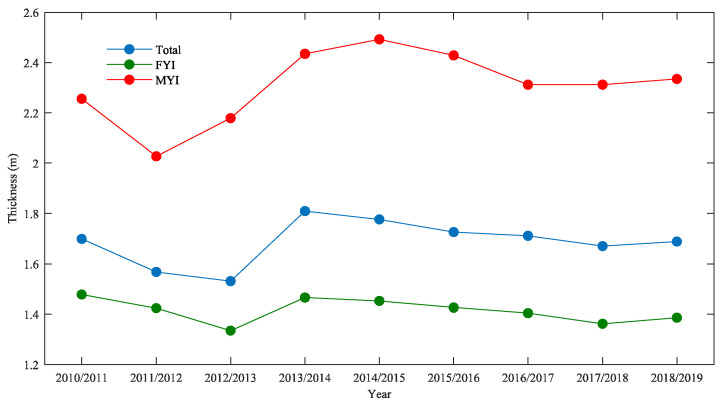
Annual average thickness variations of the Arctic sea ice from 2010/2011 to 2018/2019 estimated using the LSA method. The blue dots indicate total ice thickness, the green dots indicate first-year ice thickness, and the red dots indicate multiyear ice thickness.

Figure 7 shows the annual variations of the Arctic sea ice thickness from 2010/2011 to 2018/2019. The annual average thickness value represents the mean ice thickness during a given sea ice growth season from October (November for 2010) to April. The thickness of Arctic sea ice has undergone large inter-annual fluctuations. We can observe that MYI is the most variable ice type. Between 2010/2011 and 2011/2012, MYI thickness declined by 10% (0.229 m), followed by an increase of 23% (0.446 m) from 2011/2012 to 2014/2015 and a decrease of 6% (0.158 m) from 2014/2015 to 2018/2019. The thickness of FYI cover is much less variable than that of MYI. From 2010/2011 to 2012/2013, FYI thickness declined by 9.6% (0.143 m), followed by an increase of 9.8% (0.131 m) in 2013/2014 and a decrease of 7% (0.104 m) from 2013/2014 to 2017/2018 and a slight increase of 1.8% (0.024 m) from 2018/2019. These changes impact the total Arctic sea ice thickness, which declined by 9.9% (0.168 m) from 2010/2011 to 2012/2013, increased by 18% (0.278 m) from 2013/2014, decreased by 7.6% (0.138 m) from 2013/2014 to 2017/2018, and increased by 1% (0.017 m) in 2018/2019. The annual variation of FYI thickness is similar to the variation of total ice thickness, which is consistent with the seasonal variations. As shown in Figure 7, the mean thickness for the total sea ice during the growth season is smallest from October 2012 to April 2013. A sharp increase in total sea ice thickness in 2013/2014 can also be found in Figure 7. Kwok et al. [11] also found a notable increase in 2013 when estimating the Arctic sea ice thickness and volume with CryoSat-2 data from 2010 to 2014. Tilling et al. [12] also found a sharp increase in Arctic sea ice volume in 2013 and 2014 with CryoSat-2 data. Tilling et al. [12] pointed out that the increase was caused by the remaining thick sea ice northwest of Greenland over the summer of 2013. This is associated with the anomalous low temperature in 2013, which caused a 5% drop in the number of melting days.

### 4.3. Comparison with CryoSat-2 Sea Ice Thickness Products

**Figure 8:**
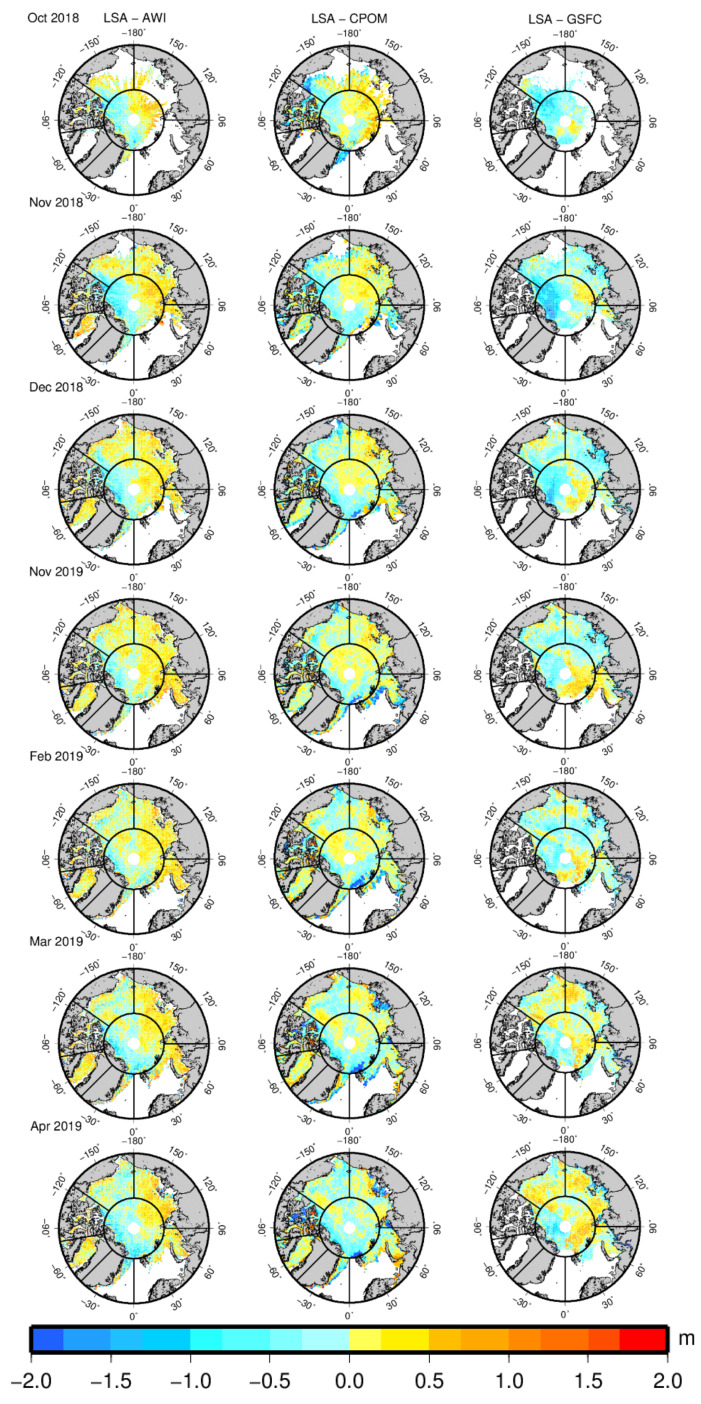
Distributions of sea ice thickness bias between the LSA and three CryoSat-2 thickness products for the 2018–2019 Arctic sea ice growth season from October to April; the left column indicates the LSA minus AWI (on 25 km grids); the middle column indicates the LSA minus CPOM (on 5 km grids); and the right column indicates the LSA minus GSFC (on 25 km grids). The bias distributions are presented for seven regions.

Comparisons between sea ice thickness estimates using the LSA method and three CryoSat-2 ice thickness products (AWI, CPOM, and GSFC) are performed for the 2018–2019 Arctic sea ice growth season. Figure 8 shows the spatial distributions of the bias between the LSA sea ice thickness and AWI, CPOM, and GSFC thicknesses. The bias distributions between the LSA and CPOM are presented on 5 km grids, while the bias distributions between the LSA and the other two products are on 25 km grids. Table 3, Table 4 and Table 5 show the mean values and RMSEs of the thickness bias between the LSA and AWI, CPOM, and GSFC, respectively, in the entire Arctic Basin and seven regions. The overall thickness from the LSA method during the growth season is higher than the thickness from the AWI and lower than the thickness from the CPOM and GSFC.

The average bias between the LSA and AWI in the entire Arctic Basin during the growth season is 0.025 ± 0.640 m. In October, the AWI product does not provide any ice thickness estimates for region 4. The mean thickness of the LSA in the entire basin is larger than that of the AWI for November through March but smaller than that of the AWI for October and April. The maximum bias (0.094 ± 0.480 m) of the two products occurs in January, and the minimum bias (0.003 ± 0.594 m) occurs in November. In regions 1, 5, and 7, the mean thickness of the LSA is smaller than that of the AWI. The bias between the LSA and AWI is less than 0.1 m in region 1 and 2. In region 1, the difference between the two products is −0.080 ± 0.494 m from 2018 to 2019. The largest mean difference in region 1 occurs in April. The bias in the western part of region 1 is positive, while it is negative in the eastern part of region 1. Region 2 features the lowest bias (0.013 ± 0.399) of the seven regions, while region 5 has the largest bias (−0.285 ± 1.048 m). In regions 3 and 4, most of the bias is positive. The bias in region 6 during October reaches −0.442 ± 1.152 m because of the limited ice in this area. The bias in regions 6 and 7 is reduced as the growth season progresses.

The overall mean difference between the LSA and CPOM is −0.143 ± 0.640 m. The largest bias (−0.173 ± 0.691 m) of the two products occurs in March 2019. Region 5 features the largest thickness bias (−0.270 ± 1.017 m) from the LSA and CPOM for the seven regions, which is consistent with the bias of the LSA and AWI. In region 1, the difference between the two products is −0.197 ± 0.525 m from 2018 to 2019. The largest mean difference in region 1 occurs in March. The bias in region 1 is positive in October and negative from November to April. The largest difference in region 2 occurs in October, and the difference is reduced as the growth season progresses. In region 3, the largest difference occurs in April. Large differences occur in southern offshore areas of region 3 during March and April. The mean ice thickness of regions 4, 5, and 6 in October is less than 0.1 m, and the difference between the LSA and CPOM is extremely large in the three regions.

The GSFC product does not provide thickness estimates in regions 5 and 6. The difference between the LSA and GSFC is −0.274 ± 0.628 m. The bias for the entire basin of the two products reaches a maximum (−0.531 ± 0.577 m) in October, reduces as the growth season progresses, and reaches a minimum (−0.118 ± 0.687 m) in April. Region 7 shows the largest bias (−0.516 ± 0.864 m) of the seven regions, while region 2 features the lowest bias (−0.167 ± 0.423 m). The bias in region 1 is −0.345 ± 0.757 m during the entire season. In October, most of the bias in region 1 is negative, whereas more positive bias appears as the growth seasons progresses, especially in the western part of region 1. This phenomenon can also be found in regions 2 and 3. Limited ice is found in region 4 during October, and the bias is extremely large (−2.144 ± 0.005 m).

From the comparison, we can find that large biases tend to occur in areas covered with thin ice, such as the margin of the Arctic Basin. Moreover, regions 4 and 5 have the lowest sea ice thickness and feature the largest bias in the comparison, which is mainly because of the limited accuracy of CryoSat-2 and other satellite altimeters for observing thin ice.

### 4.4. Validation with Coincident OIB Sea Ice Thickness

**Figure 9:**
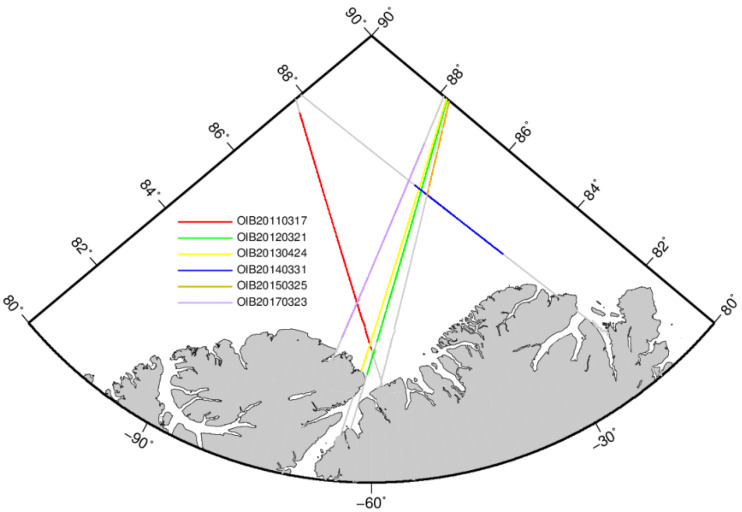
Overlapping tracks of Operation IceBridge (OIB) (colored lines) and CryoSat-2 (gray lines).

To assess our sea ice thickness estimates from the LSA method, we compare our results, as well as the three products, with the OIB sea ice thickness products. As shown in Figure 9, the ice thickness results from six OIB tracks are used for comparison. The ground tracks of the OIB are spatiotemporally coincident with the CryoSat-2 tracks. Table 6 shows the operation time of the OIB and CryoSat-2 passing times over the coincident tracks. The time differences between the two operations are within 10 h, and most of the time differences are less than 2 h. The distances of overlapping parts vary from 244 to 764 km.

**Figure 10:**
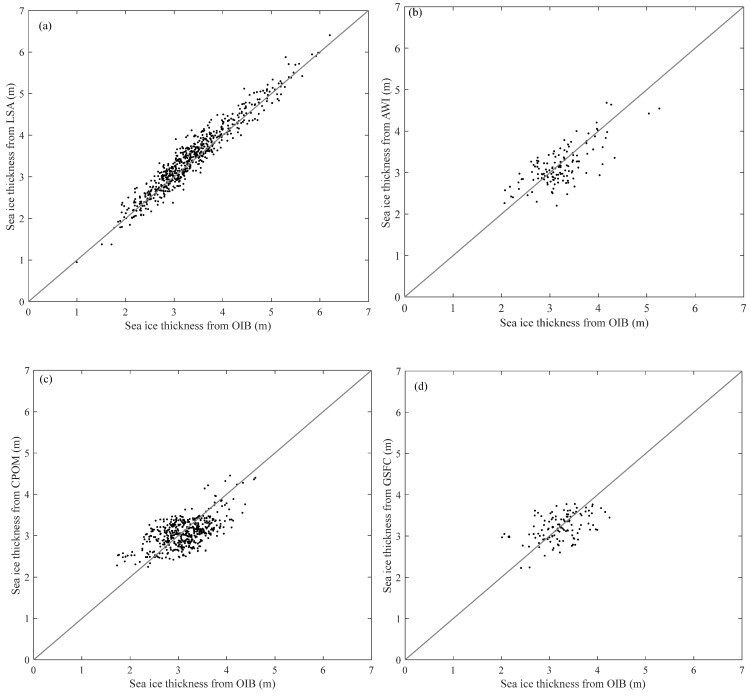
Scatterplot of the sea ice thicknesses from the LSA and the three sea ice thickness products versus those from the OIB, (**a**) LSA vs. OIB, (**b**) AWI vs. OIB, (**c**) CPOM vs. OIB, and (**d**) GSFC vs. OIB.

The overlapping parts are divided into 628 segments every 5 km, and the mean sea ice thicknesses for the LSA and OIB are calculated for each segment. While comparing with the three sea ice thickness products, the OIB thickness is averaged within a grid cell. Figure 10 shows a scatterplot of sea ice thicknesses from the LSA and the three products against OIB. Table 7 is the statistics of the comparison. The Root Mean Squared Error (RMSE) between LSA and OIB is 0.187 m, while RMSEs of the three products against OIB are larger than 0.37. Sea ice thickness from LSA also correlates well with OIB results with a correlation coefficient of 0.935. Among the three products, AWI features the highest correlation with OIB, while GSFC has the lowest correlation.

## 5. Conclusions

In this study, we develop a new method to estimate sea ice thickness from CryoSat-2 measurements without prior knowledge of parameters, including the snow depth, snow density, sea ice density, sea water density, and radar penetration factor. A quadratic model between the sea ice freeboard and thickness is established within a 5 km × 5 km grid. The model coefficients and the sea ice thickness are calculated using the LSA method. Our method can minimize the uncertainty in the conversion from freeboard to thickness.

Based on the new method, we derive estimates of the Arctic sea ice thickness from 2010 to 2019. Seasonal and annual variations of Arctic sea ice thickness are discussed. The total Arctic sea ice thickness increases each month over a given growth season from October until March or April, which is generally true for the thickness of FYI and MYI but punctuated with more variability from month to month. The seasonal and annual variations of FYI thickness are closer to the variations of total sea ice thickness than those of MYI, indicating that FYI is the dominant ice type in the Arctic sea ice cover.

To validate our estimates, we compare our results with three CryoSat-2 sea ice thickness products: AWI, CPOM, and GSFC. The comparison shows an overall difference of 0.025 ± 0.640 m for the LSA versus AWI, 0.143 ± 0.640 m for the LSA versus CPOM, and −0.274 ± 0.628 m for the LSA versus GSFC. Large differences between the LSA and the three products tend to appear in areas covered with thin ice, which indicates the limited accuracy of CryoSat-2 over thin ice. We also validated our thickness results with spatiotemporally coincident OIB thicknesses. Our results show good agreement with the OIB thickness, with a correlation coefficient of 0.935 and a difference of 0.065 ± 0.187 m.

## Figures and Tables

**Table 1:** Typical parameter values applied in previous studies.

Parameters	Values	References
Snow depth (m)	W99	[5,7,37]
	FYI: W99/2, MYI: W99	[10,27]
	FYI: AMSR-E, MYI: W99	[29,35]
Sea ice density (kg/m^3^)	925	[9]
	915	[29,30,31]
	FYI: 917, MYI: 882	[5,10,27,28]
	900	[18,32]
Snow density (kg/m^3^)	300	[32,35]
	W99	[7,36]
	320	[29,31,33]
Sea water density (kg/m^3^)	1024	[10,24,31]
	1027	[33]
	1030	[18,34]

**Table 2:** Mean ice thickness (m) of the entire Arctic Basin and seven regions for the 2018–2019 Arctic sea ice growth season.

Month	Entire	Region 1	Region 2	Region 3	Region 4	Region 5	Region 6	Region 7
October 2018	1.315	1.262	0.327	0.103	0.003	0.071	0.026	0.313
November 2018	1.454	1.592	0.521	0.311	0.078	0.127	0.259	0.473
December 2018	1.517	1.663	0.741	0.386	0.103	0.205	0.393	0.522
January 2019	1.634	1.760	0.846	0.456	0.212	0.240	0.487	0.542
Febuary 2019	1.846	1.929	1.011	0.565	0.236	0.257	0.560	0.580
March 2019	1.991	2.052	1.159	0.620	0.322	0.290	0.611	0.616
April 2019	2.056	2.164	1.167	0.615	0.291	0.259	0.590	0.586
Mean	1.688	1.775	0.825	0.437	0.178	0.207	0.418	0.519

**Table 3:** Statistics of the sea ice thickness bias between the LSA and Alfred Wegener Institute (AWI).

Statistics (m)		October 2018	November 2018	December 2018	January 2019	Febuary 2019	March 2019	April 2019	2018–2019
Entire	Mean	−0.035	0.003	0.040	0.094	0.071	0.016	−0.045	0.025
	RMSE	0.530	0.594	0.538	0.480	0.501	0.571	0.597	0.548
Region 1	Mean	−0.043	−0.107	−0.057	0.010	−0.012	−0.144	−0.200	−0.080
	RMSE	0.492	0.533	0.486	0.436	0.448	0.488	0.535	0.494
Region 2	Mean	0.010	0.004	0.086	0.100	−0.007	−0.032	−0.071	0.013
	RMSE	0.404	0.441	0.392	0.348	0.384	0.413	0.390	0.399
Region 3	Mean	0.213	0.208	0.238	0.196	0.224	0.254	0.232	0.225
	RMSE	0.412	0.373	0.310	0.285	0.349	0.380	0.518	0.377
Region 4	Mean	−	0.341	0.145	0.382	0.283	0.230	0.135	0.248
	RMSE	−	0.892	0.672	0.548	0.568	0.606	0.568	0.626
Region 5	Mean	−0.052	−0.475	−0.462	−0.301	−0.086	−0.099	−0.479	−0.285
	RMSE	0.881	0.908	1.106	0.953	1.077	1.058	1.130	1.048
Region 6	Mean	−0.442	0.333	0.204	0.195	0.175	0.195	0.130	0.192
	RMSE	1.152	0.824	0.653	0.540	0.598	0.842	0.724	0.703
Region 7	Mean	−0.308	−0.309	−0.215	−0.082	0.007	−0.103	−0.087	−0.159
	RMSE	0.784	0.824	0.776	0.751	0.700	0.798	0.861	0.792

**Table 4:** Statistics of the sea ice thickness bias between the LSA and Centre for Polar Observation and Modelling (CPOM).

Statistics (m)		October 2018	November 2018	December 2018	January 2019	Febuary 2019	March 2019	April 2019	2018–2019
Entire	Mean	−0.070	−0.169	−0.148	−0.142	−0.151	−0.173	−0.118	−0.143
	RMSE	0.634	0.607	0.566	0.609	0.651	0.691	0.681	0.640
Region 1	Mean	0.032	−0.125	−0.198	−0.137	−0.268	−0.348	−0.298	−0.197
	RMSE	0.446	0.343	0.458	0.432	0.628	0.605	0.584	0.525
Region 2	Mean	−0.278	−0.218	−0.180	−0.123	−0.084	−0.041	−0.095	−0.132
	RMSE	0.609	0.480	0.452	0.409	0.384	0.355	0.337	0.430
Region 3	Mean	0.282	0.037	0.022	0.028	−0.039	−0.204	−0.303	−0.047
	RMSE	0.358	0.410	0.390	0.335	0.443	0.665	0.836	0.549
Region 4	Mean	0.226	−0.457	−0.015	−0.339	−0.199	−0.146	0.083	−0.147
	RMSE	1.079	1.014	0.545	0.840	0.650	0.629	0.514	0.708
Region 5	Mean	−1.112	−0.174	−0.331	−0.095	−0.142	−0.307	−0.176	−0.270
	RMSE	0.834	0.706	0.903	0.841	1.011	1.133	1.182	1.017
Region 6	Mean	−0.178	−0.163	−0.129	−0.049	0.052	0.108	0.050	−0.010
	RMSE	1.200	0.693	0.600	0.537	0.466	0.543	0.529	0.574
Region 7	Mean	−0.228	−0.145	−0.124	−0.049	−0.036	0.024	−0.080	−0.083
	RMSE	0.749	0.734	0.705	0.661	0.807	0.881	0.991	0.799

**Table 5:** Statistics of the sea ice thickness bias between the LSA and the NASA Goddard Space Flight Centre (GSFC).

Statistics (m)		October 2018	November 2018	December 2018	January 2019	Febuary 2019	March 2019	April 2019	2018–2019
Entire	Mean	−0.531	−0.463	−0.348	−0.216	−0.271	−0.162	−0.118	−0.274
	RMSE	0.577	0.633	0.595	0.568	0.586	0.626	0.687	0.628
Region 1	Mean	−0.519	−0.594	−0.413	−0.205	−0.295	−0.238	−0.210	−0.345
	RMSE	0.593	0.787	0.755	0.697	0.728	0.756	0.834	0.757
Region 2	Mean	−0.493	−0.416	−0.196	−0.209	−0.206	0.002	0.063	−0.167
	RMSE	0.451	0.417	0.387	0.377	0.368	0.394	0.383	0.423
Region 3	Mean	−0.527	−0.300	−0.384	−0.332	−0.251	−0.092	−0.034	−0.234
	RMSE	0.275	0.380	0.421	0.418	0.463	0.518	0.604	0.489
Region 4	Mean	−2.144	−0.177	−0.264	0.014	−0.361	−0.341	−0.291	−0.254
	RMSE	0.005	0.437	0.446	0.564	0.568	0.571	0.606	0.575
Region 7	Mean	−0.815	−0.895	−0.680	−0.310	−0.292	−0.298	−0.326	−0.516
	RMSE	0.713	0.795	0.812	0.811	0.830	0.880	0.939	0.864

**Table 6:** OIB operation times, CryoSat-2 passing times, and distances of the overlapping tracks.

ID	OIB Operation Time	CryoSat-2 Passing Time	Distance/km
OIB20110317	17-03-2011 15:04~16:54	17-03-2011 17:30	614
OIB20120321	21-03-2012 12:22~14:13	21-03-2012 11:27	764
OIB20130424	24-04-2013 15:42~17:07	24-04-2013 15:34	721
OIB20140331	31-03-2014 14:48~15:27	31-03-2014 06:08	276
OIB20150325	25-03-2015 15:53~16:30	25-03-2015 06:42	244
OIB20170323	23-03-2017 15:54~17:04	23-03-2017 17:32	526

**Table 7:** Statistics of the comparison between sea ice thickness from LSA as well as the three products and OIB.

	LSA vs. OIB	AWI vs. OIB	CPOM vs. OIB	GSFC vs. OIB
Mean	0.065	−0.038	−0.058	0.083
RMSE	0.187	0.373	0.376	0.415
R^2^	0.935	0.716	0.610	0.462
